# Neural Efficiency and Acquired Motor Skills: An fMRI Study of Expert Athletes

**DOI:** 10.3389/fpsyg.2019.02752

**Published:** 2019-12-06

**Authors:** Lanlan Zhang, Fanghui Qiu, Hua Zhu, Mingqiang Xiang, Liangjun Zhou

**Affiliations:** ^1^Department of Leisure Sports and Management, Guangzhou Sport University, Guangzhou, China; ^2^Department of Physical Education, Qingdao University, Qingdao, China; ^3^Department of Biological Science and Medical Engineering, Beihang University, Beijing, China; ^4^Department of Sport and Health, Guangzhou Sport University, Guangzhou, China

**Keywords:** neural efficiency, motor imagery, motor representation, motor repertoire, task-specific

## Abstract

The neural efficiency hypothesis was investigated. Functional magnetic resonance imaging was used to study the differences in brain activity between athletes imagining performing different movements: basketball athletes imagined throwing and volleyball athletes imagined serving. These comparisons of brain activity among athletes imagining movements from their self-sport (e.g., a basketball throw in basketball athletes) versus movements from other sport (e.g., a volleyball serve in basketball athletes) revealed the neural energy consumption each task costs. The results showed better temporal congruence between motor execution and motor imagery and vividness of motor imagery, but lower levels of activation in the left putamen, inferior parietal lobule, supplementary motor area, postcentral gyrus, and the right insula when both groups of athletes imagined movements from their self-sport compared with when they imagined movements from the other-sport. Athletes were more effective in the representation of the motor sequences and the interoception of the motor sequences for their self-sport. The findings of present study suggest that elite athletes achieved superior behavioral performance with minimal neural energy consumption, thus confirming the neural efficiency hypotheses.

## Introduction

The difference in physiological characteristics between athletes and nonathletes is an important research field in sports science. Based on the research findings, sport industry can not only understand the physiological mechanisms of high-level athlete, but also improve athlete training and competitive performance. Neural efficiency refers to patterns of more spatially localized or less intense brain activity concomitant with equal or superior performance (Neubauer and Fink, 2009).

Compared to nonexperts, experts routinely exhibit neural efficiency when performing within their domain of expertise (Babiloni et al., 2010; Nakata et al., 2010). However, existing findings are highly variegated and are often inconsistent. Some studies showed that athletes showed increased brain activation when performing motor tasks. For example, badminton athletes showed stronger activation in mirror neuron system than novices during anticipation with videos of shuttle landing (Wright et al., 2011). Basketball athletes showed higher activity in inferior parietal lobule and inferior frontal gyrus than novices in an action anticipation task with basketball free throw (Wu et al., 2013). Expert athletes had greater neural activation than novices in somatosensory and motor planning regions when passively listening to familiar sports sounds (Woods et al., 2014). However, other studies reported that the mind of expert athletes were focused or decreased activation compared with nonexperts. For example, during mental rehearsal of archery, the premotor and supplementary motor areas, and the inferior frontal region, basal ganglia and cerebellum were active in nonarchers, whereas elite archers showed activation primarily in the supplementary motor areas (Chang et al., 2011). This focused and efficient organization of task-related neural networks was also observed in golfers during pre-shot routine (Milton et al., 2007). In another study, table tennis athletes showed less neural activation in task-related brain regions during a go/no-go visual-spatial task (Guo et al., 2017). Athletes also showed less neural activation in prefrontal cortex and insula during affective challenges than controls (Costanzo et al., 2016). These discrepancies may be related to the between-group paradigm (experts vs. novices), which entangles individual differences with neural dynamics. Thus, the use of self-reference could be a better way to probe neural efficiency.

Mentally rehearsing movements has become an important technique in sport and exercise psychology. It can be done from a first- or third-person perspective (Ruby and Decety, 2001). The first-person perspective is reported to be more embodied in the way that it involves kinesthetic representation and evokes motor simulations of one’s own body and mainly recruited the left hemisphere (Lorey et al., 2009). In this context, motor imagery is defined as a mental process involving rehearsal or simulation of a given action from a first-person perspective without overt movements (Jeannerod, 1994; Decety and Jeannerod, 1995; Hanakawa et al., 2003). Motor imagery and motor execution have been suggested to be functionally equivalent. At the behavioral level, evidence from mental chronometry suggested that it requires a similar time to imagine a movement or execute it, i.e., so-called temporal equivalence (Decety, 1996; Guillot and Collet, 2005). At the neural level, motor imagery and motor execution involve partially overlapping neural substrates (Grezes and Decety, 2001; Jeannerod, 2001; Grèzes et al., 2003). Specifically, these neural substrates are the supplementary motor area, the premotor cortex, and, in a growing number of studies, the primary motor cortex, the inferior parietal lobule, the basal ganglia, and the cerebellum (Lorey et al., 2009). Besides, complex movements such as ball games are not doable in the confined scanner. Here, the present study was intended to probe the neural efficiency hypothesis using motor imagery as an alternative to motor execution to examine functional differences in the brain activity associated with different tasks.

The anatomical substrate for motor imagery is largely mediated by the parietal-premotor cortical circuit including the inferior parietal lobule, the supplementary motor area, and the postcentral gyrus (Zhang et al., 2018). The parietal-premotor cortical circuit serves its function by building an integrated presentation of actions, objects acted on, and locations toward which actions are directed (Gallese, 2005). In particular, the inferior parietal lobule is a site at which internal models and body representations form (Macuga and Frey, 2011). The supplementary motor cortex has been reported to be involved in motor planning (Hayes et al., 2012; Marchand et al., 2013), while the postcentral gyrus is involved in somatic perceptual processes (Vidoni et al., 2010; Yamashiro et al., 2013; Arce-McShane et al., 2014). Thus, different patterns of functional activation may occur in the parietal-premotor circuit among individuals with different expertise levels while imagining the same movements.

Here, the present study was designed to resolve conflicting findings over neural consumption in sport expert domain by extending previous work in two critical respects. First, motor imagery task of expertise skills was used as test task. Since motor imagery is regarded as largely equivalent in neural level to motor execution, the neural differences found through motor imagery task is likely to reflect the true neural processes of given motor expertises. Second, self-reference and cross validation was used to test the behavioral and the neural differences. To do this, a factorial fMRI design was constructed in which basketball and volleyball athletes imagined throwing a basketball or serving a volleyball. The factorial design enabled self-reference within the group to exclude possible nuisance variables related to individual differences and cross validation between the groups to confirm the effect. These two movements were chose as experimental tasks based on two considerations. On the one hand, many components are kinematically comparable between these two movements. On the other hand, the integration of kinematical components into goal-directed action differs between them. Thus, while both groups of participants performed the same tasks, they had only professionally practiced the movements from their self-sport domain. Therefore, attenuated cortical activity (particularly in the parietal-premotor cortical circuit) along with superior behavioral performance observed while imagining movements from the self-sport compared to those observed while imagining movements from the other-sport would perfectly test the neural efficiency hypotheses.

## Materials and Methods

### Participants

Twenty-four expert basketball athletes (19.2 ± 1.4 years old, age range, 18–21 years old) and 24 expert volleyball athletes (18.9 ± 1.5 years old, age range, 17–22 years old) were studied. All participants were right-handed males (Oldfield, 1971) recruited from the basketball and volleyball teams in Shanghai University of Sport. Basketball athletes had trained 10.7 ± 1.7 h per week for 10.8 ± 1.9 years (range: 8–14 years). Volleyball athletes had trained 9.8 ± 1.3 h per week for 8.5 ± 1.1 years (range: 7–11 years). A typical training week includes 5 days of training (Tuesday to Friday and Sunday) and 2 days of rest (Monday and Saturday). The 5 days of training is made up of 3 days of professional skill training on the court (2:45–5:00) and 2 days of indoor strength training (2:45–5:00). Compared with basketball athletes, volleyball athletes trained for similar intensity as indexed by training hours [*T*(46) = 1.95, *p* = 0.06], but they started their career later as indexed by training years [*T*(46) = 5.01, *p* < 0.001]. Both groups consisted of national first- or second-level athletes who were qualified for provincial or national level competitions. The experimental procedure was approved by the local ethics committee (No. 2018126). All participants provided written informed consent prior to the experiment.

### Motor Imagery

To assess participants’ general motor imagery ability, the ease of forming visual and kinesthetic images of four basic gross movements (a knee lift, jump, arm movement, and waist bend) was measured with a Chinese translation of Movement Imagery Questionnaire-revised (MIQ-R, Hall and Martin, 1997). Participants rated their ease of imaging on a 7-point Likert-type scale ranging from 1 (very hard to see/feel) to 7 (very easy to see/feel). After the items for each subscale are averaged, a cutoff score of 5 (fairly easy) was used to ensure a participant was eligible for the following motor imagery task.

**Figure 1:**
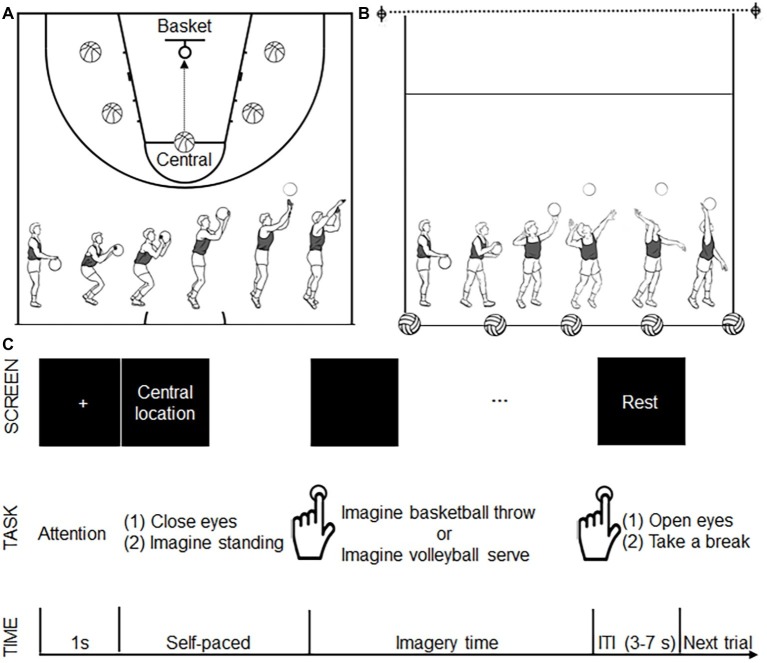
Experimental design. **(A)** The five locations (shown as a basketball) for basketball throws. All five locations were the same distance from the basket (as shown with a circle). The bottom panel shows the decomposed movements required for a basketball throw. **(B)** The five locations (shown as a volleyball) for volleyball serves. All five locations were the same distance from the net (as shown with a dotted line). The bottom panel shows the decomposed movements required for the volleyball serve. **(C)** Time course of the motor imagery trials. During each trial, after seeing a 1-s fixation cross, the participants saw a location instruction. After finishing reading the location instruction, the participants closed their eyes and imagined standing on the specified location. The participants pressed a button to signal that they had started imagining a basketball throw or volleyball serve. The participants pressed the button again when they imagined the ball had left their hand. Following the second button press, a “rest” instruction was presented on the screen, and the participants opened their eyes and took a break. This rest period served as a variable intertrial interval (ITI 3–7 s). Another fixation cross announced the start of the next trial.

Field training was applied to all eligible participants on the same day before the experiment to acquaint them with the motor tasks used in the present study, especially the motor task from the other-sport. Basketball throwing and volleyball serving were used as motor imagery tasks. Participants were instructed to use motor imagery from a first-person perspective. For basketball throwing, the participants were instructed to imagine throwing the ball toward the basket after dribbling it three times. To avoid possible fatigue or adaptation effect from a single location, five different locations each were specified for the two tasks. For basketball throwing, the five locations were the central location (the traditional point for free throws) and the other four lateral locations (bilateral to the central location) (Figure 1A). All five locations were the same distance from the basket. For the volleyball serve, the participants were instructed to strike the ball, causing it to cross the net after dribbling the ball three times. Similar to the procedure used for basketball throws, we specified five locations on the court as serve locations (Figure 1B). All five locations were the same distance from the net. The participants were instructed to perform the motor imagery task while holding the balls as a previous study revealed the importance of implementing task-related motor imagery (Wang et al., 2014).

Behavioral and fMRI measures were performed after field training on two separate days at least 2 weeks apart in a random order. The participants physically performed basketball throws or volleyball serves on the court from each of the five locations. Evidence from existing behavioral studies shows that imagined movements retain the same temporal characteristics as the corresponding executed movements (Sirigu et al., 1995). The execution process of each participant was recorded (Sony PXW-F37, 50 fps) and the durations of motor execution were obtained offline by reading the frame timer of the camcorder. The duration of motor execution was defined as the time between the onset of the first dribble and the offset when the ball left the hand. Three trials were repeated at each of the five locations (for a total of 15 trials) for both tasks. The duration of motor execution required for the total 15 trials was averaged, and the mean value was defined as the execution times for basketball throws and volleyball serves.

The motor imagery time was measured for both groups. Participants performed two runs of motor imagery, including one for basketball throws and the other for volleyball serves. The participants were instructed to mentally rehearse the motor imagery tasks from a first-person perspective with their eyes closed. The motor imagery task was self-paced. Participants pressed a button (E-prime 2.0, Psychology Software Tools Inc., Pittsburgh, PA) with the left index finger to label the beginning and end of their motor imagery task (duration of motor imagery). The instructions resulted in each of the five locations for motor imagery being presented to the participants in random order. Three trials were repeated at each of the five locations (for a total of 15 trials) for both tasks. The 15 trials for both tasks were averaged separately, and the mean values were defined as the imagery time for basketball throws and volleyball serves.

Because both motor imagery and execution are likely to be constrained by the same physical laws (Decety and Jeannerod, 1995; Decety, 1996), a difference in the time required for motor imagery and motor execution tasks may indicate the effects of other physiological factors on the measurement (Sirigu et al., 1995, 1996). Temporal congruence was used as a valid mental chronometry index to evaluate functional equivalence between motor imagery and motor execution (Guillot and Collet, 2005). In the present study, variances in motor time (for both execution and imagery) between the two participant groups were dependent on differences in expertise levels in the self- versus other-sport. The temporal congruence was calculated in the same manner as did in Zhang et al. (2018). A lower ratio score indicates better congruence between execution time and imagery time. Temporal congruence was calculated for each group on each task separately.

### Functional Magnetic Resonance Imaging

The experiment was performed using a 2-group (basketball athletes, volleyball athletes; between-participant factor) × 2 task (basketball throw vs. volleyball serve; within-participant factor) factorial design. The experiment consisted of two runs: imagining a basketball throw with a basketball and imagining a volleyball serve with a volleyball. The order of the two runs was counterbalanced among the participants. Each run lasted 6 min, during which 180 volumes were acquired. Each run consisted of 25 trials (5 trials at each location presented in a random order). The participants watched a screen *via* a mirror mounted on the MRI head coil. Time course of the motor imagery trial was illustrated in Figure 1C. A 1-s fixation point appearing at the center of the screen indicated the start of a trial. The instruction for one of the five locations was subsequently presented to the participant. Participants pressed a button to mark the beginning and end of their motor imagery (E-prime 2.0, Psychology Software Tools Inc., Pittsburgh, PA). Two adjacent trials were separated by a resting state indicated by a black screen that lasted 3–7 s after the second button press (end of motor imagery). The resting state was used as a baseline measurement. Imaging was acquired using a 3.0 Tesla Siemens Trio Tim MRI scanner with a 12-channel head coil at Shanghai Key Laboratory of Magnetic Resonance. Functional images (repetition time = 2 s, one shot per repetition, echo time = 30 ms, flip angle = 90°, field of view = 240 mm^2^ × 240 mm^2^, slice thickness = 4 mm, voxel size = 3.3 mm^3^ × 3.3 mm^3^ × 4 mm^3^, slices per volume = 33, volumes for 6 min = 180) were obtained as a gradient echo planar imaging sequence.

### Post-scanner Imagery Questionnaires

Immediately after the participants were released from the scanner, the performance of motor imagery tasks was measured with self-evaluation using two questionnaires. The first questionnaire was developed based on that used in Wang et al., 2014 to assess if participants adhered to the experimental protocol. It included four introspective items and was scored on a 5-point Likert-type scale range from 1 (not at all) to 5 (greatly). The first question asked the participants to what extent they have used a first-person perspective during motor imagery. The second question asked the participants to what extent the motor imagery was easily controlled. The third question asked to what extent the first-person perspective motor imagery was clear. The fourth question asked to what extent the ease of performing first-person perspective motor imagery was different between the tasks (basketball throw vs. volleyball serve). The second questionnaire tested the vividness of motor imagery, was developed based on the MIQ-R (Hall and Martin, 1997) and included eight questions related to kinesthetic and visual properties (4 for each) during the motor imagery task. They were rated on a 7-point Likert-type scale ranging from 1 (very hard to see/feel) to 7 (very easy to see/feel).

### Data Analysis

#### Mental Chronometry Test

Temporal congruence was tested with a two-way repeated measure analysis of variance (ANOVA), with the groups (basketball athletes and volleyball athletes) used as the between-participant factor and the tasks (basketball throw, volleyball serve) used as the within-participant factor.

#### Post-scanner Imagery Questionnaires

The vividness of motor imagery data was also analyzed with two-way repeated measure ANOVA. The use of first-person perspective motor imagery was tested with unpaired *t* test.

#### Functional Magnetic Resonance Imaging

The imaging data were preprocessed and analyzed with a general linear model (Friston et al., 1994) using Statistical Parametric Mapping version 8^1^ implemented in MATLAB R2013a (MathWorks, Inc., Natick, MA). Preprocessing included slice time correction, realignment, normalization, and spatial smoothing in sequence. Normalization was performed by directly registering the mean functional image to the standard Montreal Neurological Institute template provided by SPM8. The resulting interpolated spatial resolution was 3 mm^3^ × 3 mm^3^ × 3 mm^3^. The functional data were then smoothed with a Gaussian kernel of 6-mm full-width at half-maximum.

The first-level analysis was computed within-participant using an event-related approach in the context of the general linear model. Statistical parametric *t*-maps were generated for each participant. For each participant, the regressor of interest was defined to characterize cerebral responses to imagery for the four different conditions in the 2 × 2 factorial design. A regressor of no interest was used to model the cerebral responses to a button press. For the motor imagery regressor, onsets were time-locked to the button press that marked the onset of motor imagery, and durations corresponded to the mean motor imagery durations across all imagery trials of the participant. To address the effects of potential contamination by button pressing (Eden et al., 1999), button pressing was included as a condition in the design matrix to model the cerebral responses to button presses. In this way, the regressor of motor imagery would not pick up cerebral responses induced by button press, and this enabled us to regress out the effect of button presses (Bakker et al., 2008). For the button press regressor, onsets were time-locked to the time point each button press was made, and the duration was set at zero. Each effect was modeled on a trial-by-trial basis. Also, the head motion regressors derived from the spatial realignment procedure were included. The rest period was used as the baseline. The contrast images for cerebral responses to motor imagery were compared with those for the rest period and stored for a second-level group analysis.

For second-level group analysis, an SPM8 full factorial model was constructed using a two-way ANOVA model to test whether there were regions showing differences between the two participant groups (between-participant factor) and whether the differences in these regions between the groups were task-dependent. Contrast values for significant clusters were extracted from individual data obtained under the two experimental conditions to conduct correlation analyses. For each group, for each of the two experimental conditions, the mean contrast values and standard errors were calculated to characterize whether the pattern of interaction constituted an effect of expertise. Because there was variance in the duration of motor imagery time (Table 1) and that blood oxygenation level dependent (BOLD) signal changes occurred linearly with the duration of neural responses, the mean duration of motor imagery across all trials for each participant was added as a nuisance covariate to ensure that the main results were not affected by interparticipant variation (Dale and Buckner, 1997). Similarly, to make sure the comparability between tasks, the vividness of motor imagery of each participant was also added as a nuisance covariate. The false discovery rate (FDR) approach was used to correct for multiple comparisons (Genovese et al., 2002) at the cluster level with an extent threshold of 15 voxels per cluster (*p* < 0.05). All statistical maps were overlaid on the CH2 template (Holmes et al., 1998).

**Table 1:** Duration of motor imagery.

Group	Basketball throw	Volleyball serve
Basketball athletes	4.17 ± 0.22 s	4.18 ± 0.19 s
Volleyball athletes	4.89 ± 0.15 s	4.54 ± 0.16 s

*The durations of motor imagery shown in this table were measured during fMRI scanning. The values are expressed as the mean ± standard error of the mean*.

#### Parameter Analysis

For each significant cluster, the Pearson’s correlation was tested for all athletes between the behavioral measure (the difference in the temporal congruence of motor imagery between self-sport imagery and other-sport imagery) and the functional measure (the difference in the contrast value between self-sport imagery and other-sport imagery). The threshold for significance was set at *p* < 0.05.

## Results

### Expertise-Dependent Behavioral Performance During Motor Imagery

**Figure 2:**
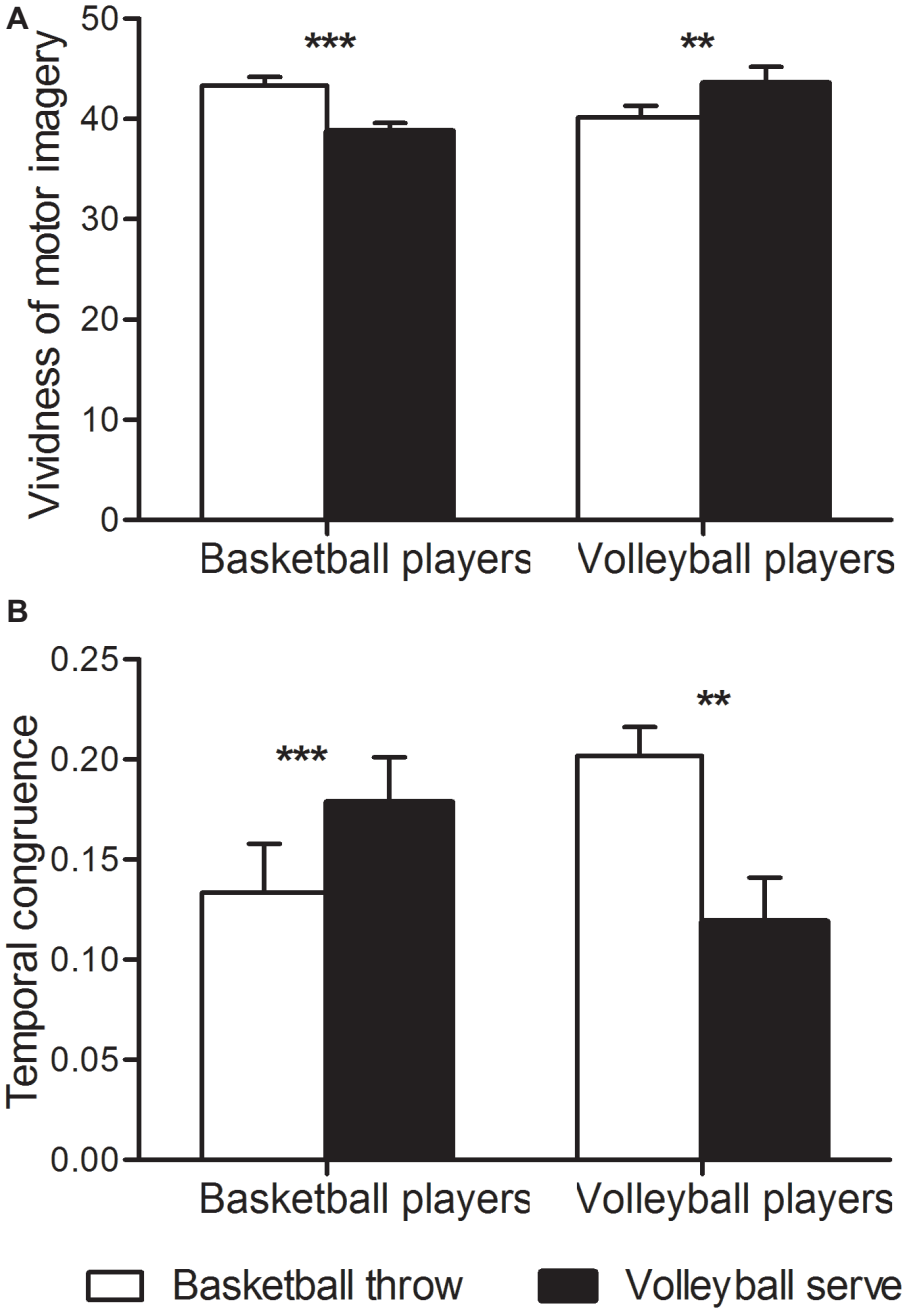
Behavioral measures. **(A)** Temporal congruence. The ordinate shows temporal congruence. Congruence is expressed as [imagery time] minus [execution time] divided by [imagery time plus execution time]. **(B)** Vividness of motor imagery. ^**^p < 0.01; ^***^p < 0.001.

A two-way analysis of variance of temporal congruence revealed a significant interaction between group and imagery task [*F*(1, 46) = 23, *p* < 0.001]. Further *t*-tests revealed that in basketball athletes, congruence between motor imagery and motor execution was higher when imagining a basketball throw than when imagining a volleyball serve (*p* < 0.001), whereas in volleyball athletes, congruence was higher when imagining a volleyball serve than when imagining a basketball throw (*p* < 0.01) (Figure 2A).

According to the scores on the questionnaire aimed at examining first-person motor imagery, both groups performed the motor imagery task with a first-person perspective without group difference [basketball athletes, 14.92 ± 2.48; volleyball athletes, 15.79 ± 1.92; *T*(46) = −1.37, *p* = 0.18].

Two-way analysis of variation of the vividness of motor imagery revealed a significant interaction between group and imagery task [*F*(1, 46) = 46, *p* < 0.001]. Further *t*-tests revealed that basketball athletes imagined basketball throws more clearly than they imagined volleyball serves (*p* < 0.001), whereas volleyball athletes imagined volleyball serves more clearly than they imagined basketball throws (*p* < 0.01) (Figure 2B). No significant main effects were found for group or task.

### Expertise-Dependent Functional Activation of Motor Imagery

**Figure 3:**
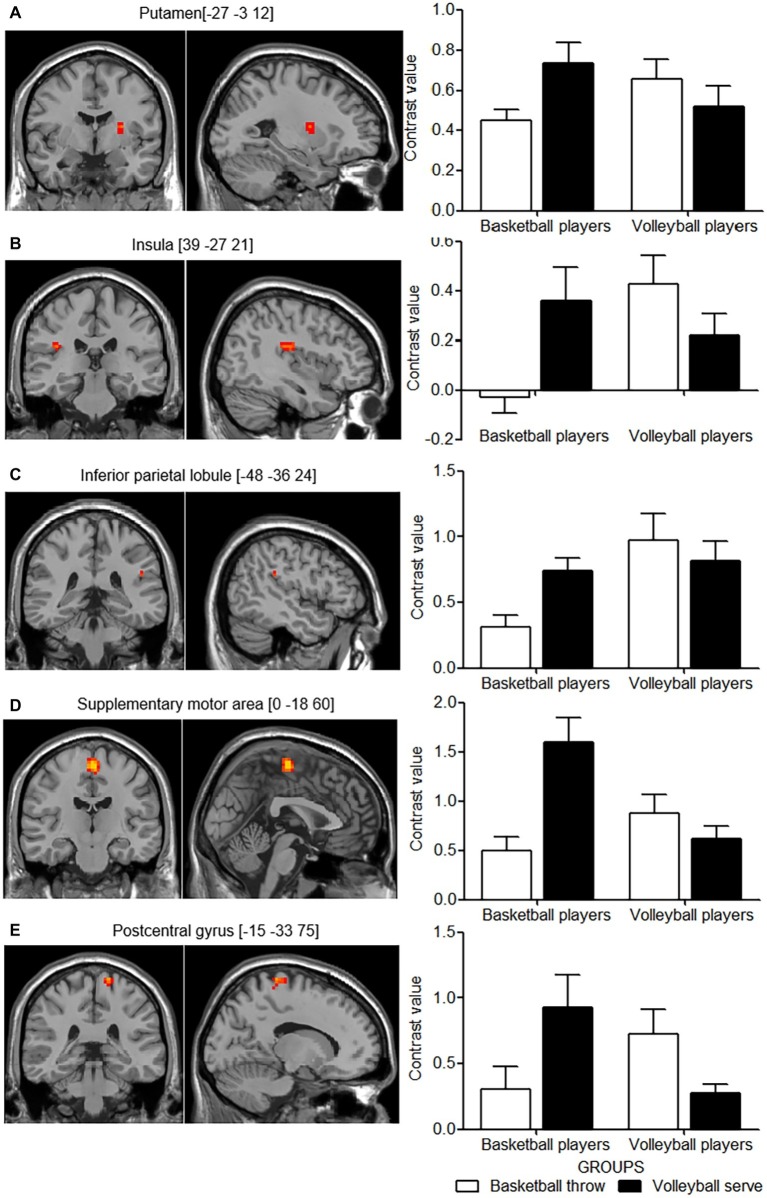
Cortical areas showing interactions between group and motor imagery tasks. The left panel shows the cortical areas with activation in axial (z) and sagittal (x) views based on the interaction effect for group and motor imagery task. The right panel shows the mean contrast value for each activation cluster for each group in the two imagery tasks. **(A**–**E)** show the pair of left and right panels for each activation cluster of the interaction effect. White columns indicate basketball throws, and black columns indicate volleyball serves. Error bars indicate the standard error of the mean.

The differences in cortical activity between the two imagery tasks in the two groups of expert athletes were of more interest. *F* contrast revealed an interaction between these two main factors in the left putamen, the right insula, the left inferior parietal lobule, supplementary motor area and postcentral gyrus (Table 2, Figures 3A–E, left panel). The significant interaction was further investigated by examining the contrast values in the parameter estimate. Expert athletes showed less activation when imagining movements from their self-sport than when imagining movements from the other-sport, and there was a crossover pattern for the interaction between imagery task and group (Figure 3, right panel).

**Table 2:** Expertise effect on motor imagery.

Brain area	Side	Cluster	MNI coordinate (mm)			*F*
			x	y	z	
Putamen	L	16	−27	−3	12	24.58
Insula	R	47	39	−27	21	25.28
Inferior parietal lobule	L	49	−48	−36	24	34.06
Supplementary motor area	L	125	0	−18	60	32.98
Postcentral gyrus	L	31	−15	−33	75	27.96

*L = left. FDR-corrected to p < 0.05, extent threshold 15 voxels*.

### Parameter Analysis

Correlation analysis between behavioral measures and functional measures revealed a significant negative correlation in the left inferior parietal lobule (*r* = −0285, *p* = 0.049). No significant correlation was found for the left supplementary motor area (*r* = −0.218, *p* = 0.137), postcentral gyrus (*r* = −0.129, *p* = 0.382), putamen (*r* = −0.143, *p* = 0.331) and the right insula (*r* = −0.043, *p* = 0.769).

### Discussion

The present study tested the neural efficiency hypotheses. Results showed that motor imagery performance was superior but cortical activation was decreased in athletes during imagining movements from their self-sport, suggesting a relatively facilitated motor simulation process for self-sport.

### Expertise-Dependent Behavior of Motor Imagery

At the behavioral level, athletes showed better congruence in the time course between motor execution and motor imagery and greater vividness of motor imagery for the self-sport than for the other-sport. Both groups significantly overestimated the motor imagery duration for movements associated with the other-sport. This difference could be attributed to the extra effort required to represent the detailed movement components of unfamiliar movements (Guillot and Collet, 2005). The superior behavioral performance achieved in the self-sport indicated the presence of effective internal motor representation processes related to the self-sport (Guillot and Collet, 2005). Aspeculation is that this process is the mechanism underlying the effect of neural efficiency. This will be examined further in the next section from the neural representation level.

### Expertise-Dependent Functional Activation of Motor Imagery

The experiment’s factorial design revealed an interaction effect for group and task in the parietal-premotor cortical circuit. Besides, the left putamen and the right insula also showed the interaction effect. Neural activation in these regions was attenuated for the self-sport imagery compared with that for the other-sport imagery. This difference was interpreted as evidence of neural efficiency, i.e., motor imagery performance was superior for the self-sport, which required less energy consumption.

The findings of present study are consistent with those of a previous study which showed that there was left hemisphere dominance during the simulation of hand movements from a first person perspective (Lorey et al., 2009). This confirmed the embodied nature of the motor imagery paradigm used in the present study (i.e., the first-person perspective). This embodied process has in particular been associated with the left inferior parietal lobule. The inferior parietal lobule is a site associated with internal models and body representations (Macuga and Frey, 2011) that has been reported to be recruited when observing kinematic display of one’s own movements (Bischoff et al., 2012) and during first-person perspective motor imagery (Lorey et al., 2009). The embodied simulation processes that occur in the inferior parietal lobule have also been emphasized by other studies (Keysers et al., 2004; Gallese, 2005). Furthermore, the inferior parietal lobule is related to the degree to which an action is embodied (Cross et al., 2006). The decreased activation during self-sport imagery is likely because that the internal representation of self-sport movements configured with the athletes’ motor reservoirs that are shaped by long-term training and overpractice requires less neural resources to simulate the task. The inferior parietal lobule is also involved in pragmatic analysis related to action-oriented object manipulation (Jeannerod, 1994; Buccino et al., 2001) and codes for specific goals or intentions of motor acts (Fogassi et al., 2005). Together with the premotor areas, the parietal-premotor cortical circuit functions to build an integrated presentation of actions, objects acted on and locations toward which actions are directed (Gallese, 2005). Both tasks performed in the present study required participants to integrate their kinesthetic movements with their manipulation of the ball and to aim the ball at the target (basket/net). However, participants were more skilled in performing the integrated representation of the self-sport, which may have involved more efficient motor simulation and less neural effort.

The supplementary motor cortex is reported to be involved in motor planning, especially for the orderly performance of complex motor sequences (Hayes et al., 2012; Marchand et al., 2013). Patients with left supplementary motor area disorders showed impaired procedural learning (Ackermann et al., 1996). The activation in the supplementary motor area was also relatively lower when participants imagined movements from their self-sport relative to when they imagined movements from the other-sport. This finding is in accordance with that of Milton et al. (2007), who found that the level of supplementary motor area activity was lower in expert golfers than in novices. In the present study, both imagery tasks required coordination of different body parts to achieve a series of movements that were performed in a well-organized temporal order. Additionally, the supplementary motor area has been reported to play a leading role in the action-monitoring system that assesses ongoing actions and detects errors (Bonini et al., 2014) and to be involved in more extensive executive control activities, including reducing inference from irrelevant, distracting features in the environment (Nachev et al., 2008). The level of activation in the supplementary motor area was lower during imagery related to the self-sport, suggesting that the orderly representation of the movement components meant less effort and neural cost were needed (Shiffrin and Schneider, 1977).

The postcentral gyrus is classically associated with somatosensory processing (Iwamura et al., 1994; Tamè et al., 2016). This area is recruited even when athletes passively listen to sports sounds (Woods et al., 2014). Similar to the inferior parietal lobule and the supplementary motor cortex, the activation of the postcentral gyrus was also down-modulated for the self-sport. According to the somatosensory homunculus, this activation cluster was the trunk area. Trunk muscle function plays an important role in both sports but kinematic chains of the trunk differ (da Silva Santos et al., 2017; Sekiguchi et al., 2017). It is plausible that athletes from one domain were clearly aware of the different kinematic chains of the two different tasks and made different performance of them. Taken together, participants were able to generate automatic motor procedures related for self-sport as a result of long-term training. This may allow them to complete the motor task with least assistance from somatosensory inputs, resulting in a decrease in the activation of the postcentral gyrus. A similar reduction in cortical activation in the postcentral gyrus was observed in pianists, who may suppress sensory feedback to enable smooth and sequential motor behaviors (e.g., shifting from one key to another) (Oechslin et al., 2012).

Putamen, as part of the basal gangalia, supports willed, intentional movements (Groenewegen, 2003). In particular, putamen has been reported to be important for chucking movement sequences by concatenating movements at various stages (Wymbs et al., 2012). Chunking allows performance of a well learned motor sequence to be executed as a single unit of activity rather than multiple individual actions. Thus, decreased activity in putamen for self-sport imagery may reflect a more effective (chunked) representation that accompanies automatization as a result of extensive training for self-sport. Our findings were in line with a previous study which found training-related decrease in putamen even after a short time learning for a motor sequence task (Poldrack et al., 2005).

The right insula has been reported to be the seat of interoception (Critchley et al., 2004) or part of the pathway of interoceptive awareness (Khalsa et al., 2009). Either way, the right insula seems to play an important role in generating accurate predictions of the bodies’ internal state in the next moment. The model of the body’s future condition further instructs other brain areas to initiate actions that are more tailored to coming demands (Faull et al., 2018). It is plausible that predicting interception during other-sport imagery is more challenging than that of self-sport imagery, thus needs more efforts (increased activity in the right insula).

### Relationship Between Behavioral and Functional Measures

A negative correlation was found between the behavioral measure and the functional measure for elite athletes. This finding suggests that greater neural efficiency underlies superior motor performance. In other words, better behavioral performance at less neural effort enables individuals to perform the internal processes associated with movements of high proficiency. This is plausible because well-established skills are believed to be based on automaticity and reduction in nonessential inputs of task-irrelevant processes (Logan and Crump, 2009; Rieger, 2012). Attention to such automatic processes can even undermine performance (Beilock et al., 2002). Neural efficiency may stem from the long-term training and be task-specific which enabled athletes to develop a focused and efficient organization of task-related neural networks.

### Limitations

Our findings should be interpreted in light of the following limitations. The cross-sectional design of the present study, though articulate that neural efficiency is task-specific, is insufficient to impute that neural efficiency is training-induced. Further studies that randomly allocate naive participants to different sport interventions are expected to separate the effects of training and other confounding factors. Additionally, the correlation between behavioral measure and functional measure, although existed, was weak.

## Conclusion

The present study revealed greater temporal congruence and vividness of motor imagery but attenuated activity in the parieto-premotor motor representation circuit and the insula interoceptive cortex when athletes imagined movements from the self-sport compared with when they imagined movements from the other-sport. Athletes were more effective in the representation of the motor sequences and the interoception of the motor sequences for their self-sport. The finding that elite athletes achieved superior behavioral performance with minimal neural energy consumption thus confirms the neural efficiency hypotheses.

## Data Availability Statement

The datasets generated for this study are available on request to the corresponding author.

## Ethics Statement

The studies involving human participants were reviewed and approved by the ethics committee of Shanghai University of Sport. The patients/participants provided their written informed consent to participate in this study.

## Author Contributions

LZha concepted, drafted, and revised the work. FQ and HZ acquired and analyzed the data. LZho and MX concepted the work, intepreted the data, and revised the manuscript critically for important intellectual content. All authors approved the final version and agreed to be accountable for all aspects of the work.

### Conflict of Interest

The authors declare that the research was conducted in the absence of any commercial or financial relationships that could be construed as a potential conflict of interest.

## Footnotes

^1^http://www.fil.ion.ucl.ac.uk/spm
